# Antimicrobial photodynamic therapy using a low-power 650 nm laser to inhibit oral *Candida albicans* activity: an in vitro study

**DOI:** 10.25122/jml-2023-0285

**Published:** 2024-01

**Authors:** Roaa Osamah Adnan, Hussein Ali Jawad

**Affiliations:** 1 Institute of Laser for Postgraduate Studies, University of Baghdad, Baghdad, Iraq

**Keywords:** antimicrobial agents, *Candida albicans*, diode laser, endodontic failure, methylene blue, oral cavity, photodynamic therapy, photosensitizer, 650 nm

## Abstract

This study assessed the efficacy of antimicrobial photodynamic therapy (PDT) using a 650 nm diode laser combined with methylene blue (MB) as a photosensitizer to inhibit the growth of *Candida albicans (C. albicans)*. Oral samples were collected from 75 patients diagnosed with oral thrush. *C. albicans* was isolated and identified using traditional methods and the VITEK 2 YST system. Samples (*n* = 25) were divided into five groups: Group 1 (control, *n* = 5) consisted of *C. albicans* suspensions in saline; Group 2 (*n* = 5) treated with nystatin; Group 3 (*n* = 5) exposed to a 650 nm diode laser in continuous mode at 200 mW for 300 seconds; Group 4 (*n* = 5) treated with 650 nm laser and MB as a photosensitizer; Group 5 (*n* = 5) exposed to the laser in combination with nystatin. Statistical analysis using ANOVA, Dunnett's t-test (*P* = 0.05), and LSD (*P* = 0.001) revealed significant differences in *C. albicans* counts pre- and post-treatment. Group 5 showed the most significant reduction in *C. albicans*, followed by Group 4, while Groups 2 and 3 showed the least variation. The findings suggest that PDT using a 650 nm diode laser with methylene blue (in continuous mode at 200 mW for 300 seconds) effectively reduced the prevalence of *C. albicans*.

## INTRODUCTION

The oral cavity harbors a variety of microorganisms, including viruses, bacteria, fungi, and protozoa, creating an environment suitable for the proliferation of several microbial species, which, under certain conditions, can become pathogenic [1,2]. Among these, *Candida* infections in the oral mucosa have become increasingly prevalent. Most oral fungal infections are attributed to *Candida albicans (C. albicans)*, accounting for 50% to 70% of cases. Other *Candida* species, such as *Candida glabrata, Candida tropicalis, Candida parapsilosis*, and *Candida krusei*, also contribute to these infections [3-6].

It has been observed that fungi can survive within various parts of the oral cavity, including root canals, dentine walls, and periodontal pockets [7-10]. The majority of *C. albicans* infections are linked to the formation of biofilms resistant to conventional antimicrobial treatments. Consequently, underlying fungal and bacterial contamination is likely to play a role in endodontic or periodontal treatment failures [11-16].

*Candida albicans* is a common commensal microorganism in the oral cavity of healthy individuals. However, it can potentially become pathogenic and cause candidiasis, particularly in individuals with weakened or compromised immune systems. Under certain circumstances, such as immunodeficiency or other predisposing factors, *C. albicans* can transition from a harmless commensal to an aggressive pathogen, leading to candidiasis and related oral health issues [17-20]. The treatment of candidiasis is often challenging, but it can be addressed effectively with a personalized and comprehensive clinical assessment to identify the underlying primary or secondary causes of the condition. Managing mycosis (fungal infection) involves addressing multiple risk factors simultaneously. Therefore, it is crucial to conduct a mycological examination, coupled with antifungal susceptibility testing, followed by the initiation of antifungal therapy. This approach ensures a more effective and tailored treatment strategy for candidiasis [4-6,17]. To minimize the risk of recurrent candidiasis and the emergence of drug-resistant yeast strains, it is advisable to maintain a treatment duration of at least three to four weeks. This precautionary measure is essential to mitigate potential challenges arising from insufficient treatment duration or incorrect dosage administration. Additionally, comprehensive treatment should encompass both the oral mucosa and, if applicable, denture plates [6,17].

Currently, no antifungal medication can effectively combat the broad spectrum of pathogenic characteristics displayed by different fungal species. The growing resistance of *Candida* species to antifungal drugs is a significant challenge in modern medical practices, underscoring the necessity to develop innovative approaches for treating oral candidiasis (OC) [3]. Antimicrobial photodynamic therapy (PDT) is a potential alternative to antifungal drugs [21]. Antimicrobial photodynamic therapy depends on the interaction of three components: a photosensitizer, a light source with a wavelength corresponding to its maximal absorption [22], and oxygen molecules. The stimulated photosensitizer initiates a cascade of chemical reactions that produce reactive oxygen species that destroy pathogenic microorganisms. Recently, PDT has been integrated into various dental practices, including as an adjunctive method for root canal disinfection in endodontics, enhancing general dentistry and periodontal treatments [23,24]. However, the literature lacks comprehensive studies on the efficacy of combined laser therapy and antifungal agents, such as nystatin, in fungal infections. This research aimed to investigate the efficacy of a specific laser wavelength (650 nm) in conjunction with photodynamic therapy for inhibiting *C. albicans* colonies.

## MATERIAL AND METHODS

This study was conducted at the Institute of Laser for Postgraduate Studies, University of Baghdad, Baghdad, Iraq. The fungal samples were collected from patients and cultured on selective media to isolate *C. albicans*.

### Sample collection and preparation

*Candida albicans* strains were isolated from the oral cavities of patients diagnosed with oral thrush, following the protocol described by Martín *et al*. [25]. These isolates were provided by the Department of Basic Science and Microbiology at the University of Baghdad, specifically for evaluating the antifungal properties of the compounds under study. The strains were cultured on Sabouraud Dextrose Agar and incubated at 37°C for 48 hours to promote growth.

**Figure 1:**
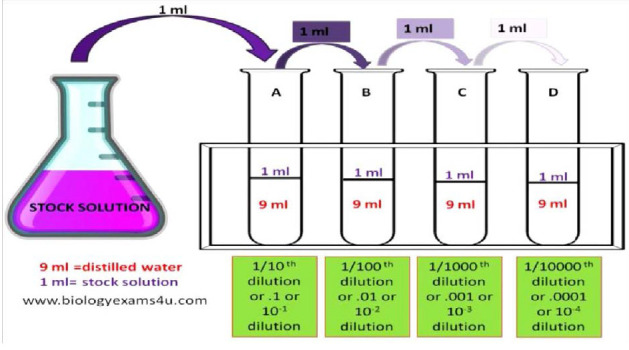
Serial dilution (Reproduced from biologyexams4u.com)

A serial dilution was performed 24 hours post-incubation to determine the optimal concentration for subsequent culturing. This process involved diluting the original 10 mL fungal culture by transferring 1 mL to a test tube containing 9 mL of distilled water, a procedure repeated across three additional tubes to achieve a range of dilutions (Figure 1). Swabs from each dilution were cultured to identify the concentration suitable for further experimentation. Colony-forming units (CFU) ranging from 30 to 300 were considered. Samples diluted by 10^-4^ resulted in 164 CFU on the agar media (Figure 1), while other samples had CFU counts exceeding 30 and were excluded.

Following the isolation of *Candida albicans* strains, a streaking technique was utilized on Sabouraud Dextrose Agar (SDA) to obtain pure fungal colonies. These agar plates were then incubated for 24 hours at 37°C. The suspension was diluted using an optical density of 0.5 McFarland standard solution. The concentration of *C. albicans* prepared was 10^-4^ viable cells/mL. For the experiment, a suspension of *C. albicans* (at a concentration of 10^-4^ cells/mL) was transferred into the wells of a 96-well microplate containing 0.1 mL of the suspension. The diameter of the cultivation area for every well was 0.36 cm.

Methylene blue (MB), used as a photosensitizer, was prepared as a stock solution at 300 µg/mL concentration. This stock solution was made using a sterile saline solution with a pH of 7.8. Consequently, the working concentration of MB in the experiment was adjusted to 100 µg/mL. Before laser exposure, *C. albicans* suspensions were pre-incubated with the MB solution for 5 minutes. During this time, 0.1 mL of liquid at room temperature was added to the wells. All antifungal activity tests were conducted under sterile conditions, within a laminar flow hood, at room temperature, and in complete darkness to ensure the absence of external light influences.

## Experimental design

### Determination of optimal laser power

A preliminary assessment was conducted to determine the optimal exposure duration required to inhibit fungal growth effectively. After testing intervals of 100, 150, 200, 250, and 300 seconds, a duration of 300 seconds was identified as the most effective for achieving significant fungal inhibition. In addition, to identify the optimal power setting for the 650 nm diode laser (Woodpecker LX 16 Plus) that could maximally reduce the CFU/ml of *Candida albicans*, another preliminary assessment was conducted. We assessed the antifungal efficacy of various power settings over a consistent irradiation duration of 300 seconds. The laser powers tested included 50 mW, 100 mW, 150 mW, and 200 mW. The 200-mW setting was the most effective in reducing the CFU/ml of *Candida albicans*, establishing it as the optimal power for subsequent experiments.

### Study group

This study contained five groups. A total of 25 samples were included in the study, with each group consisting of 5 samples. Following exposure to the antifungal agent, all samples within each group were subjected to CFU/mL counting. The fungicidal efficiency of each agent was evaluated.

**Figure 2:**
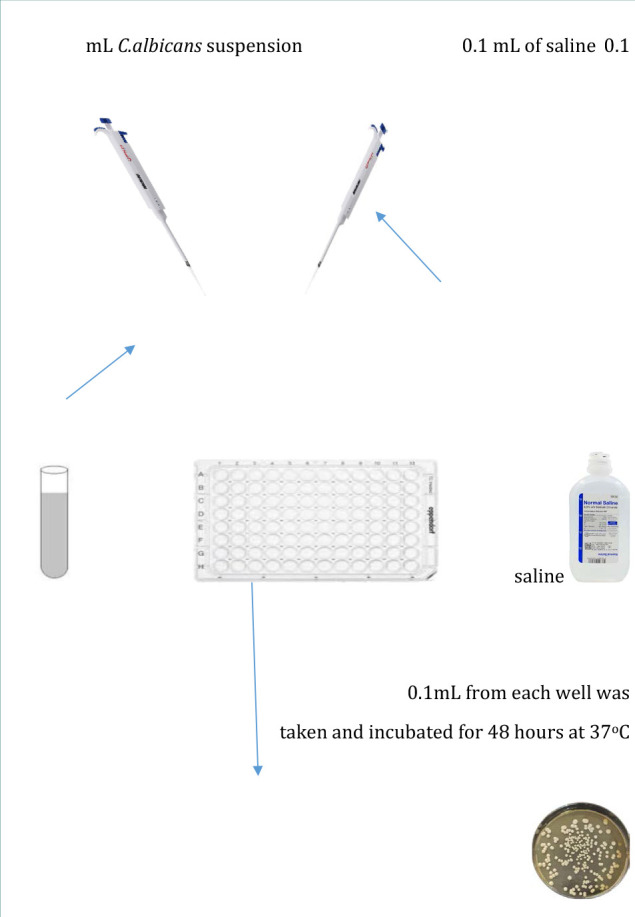
Schematic representation of procedures in Group 1 (control group)

**G1 (Control, *n* = 5)**: No treatment was administered, serving as the baseline for comparison (Figure 2).

**Figure 3:**
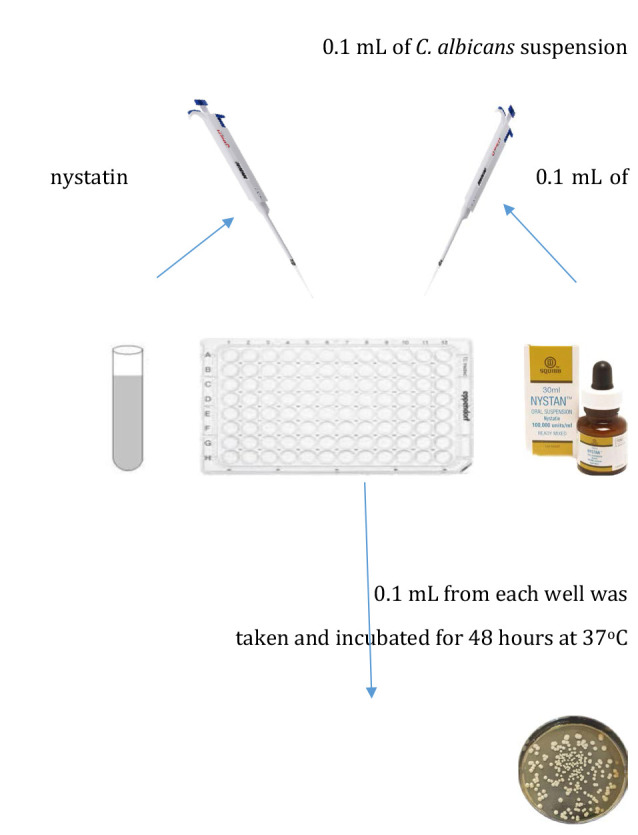
Schematic representation of nystatin treatment in Group 2

**G2 (*n* = 5)**: Samples were treated with nystatin alone (Figure 3).

**Figure 4:**
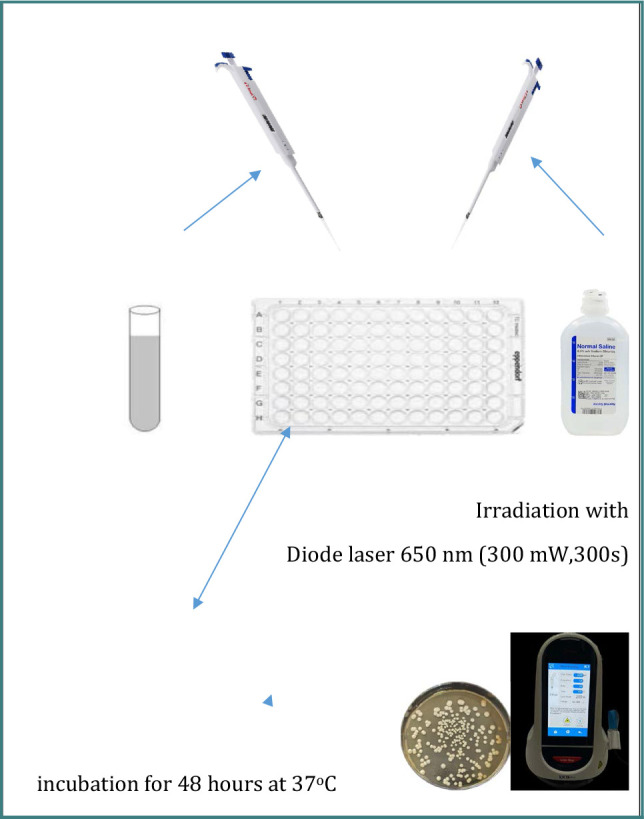
Schematic representation of diode laser irradiation in Group 3

**G3 (*n* = 5)**: Samples were exposed to a 650 nm diode laser at 200 mW for 300 seconds (Figure 4).

**G4 (*n* = 5)**: Samples received 650 nm diode laser treatment (200 mW, 300 seconds) with MB as a photosensitizer.

**G5 (*n* = 5)**: Samples received diode laser treatment (650 nm, 200 mW, 300 seconds) and nystatin.

For the experiment, 0.1 mL of the *Candida albicans* suspension was added to the wells of a 96-well microplate. To establish the control, five wells (Group 1) were left untreated.

### Antifungal activity test

0.1 mL of a diluted fungal culture containing 1×10^-4^ CFU/mL was administered to Group 2 along with 0.1 mL of nystatin. Following that, 0.1 mL of the mixture was seeded onto SDA plates. The dishes were then incubated for 48 hours at 37°C. The number of total *Candida albicans* colonies (expressed as colony-forming units, CFU/mL) was used to analyze the plates. A sample of 30 and 300 colonies was chosen for counting using the Swanson, Petran, and Hanlin approach [26].

### Irradiation procedure

The diluted mixture (1×10^-4^ cell/mL) was moved to a 96-well microplate (0.1 mL in each well) using an automated sampler. Each of these procedures was conducted within a sterile and light-restricted laminar hood. The fungal suspension was exposed to different lasers with specific settings. The irradiation took place at a normal incidence angle, ensuring that the laser beam remained perpendicular to the entrance of the wells.

### Statistical analysis

The data was analyzed using SPSS software, version 20. A one-way analysis of variance (ANOVA) test was conducted to compare mean values and standard deviations (SD) among different groups. Statistical significance was determined at a *P* value of 0.05. To examine differences between the tested groups and the control group, Dunnett's multiple comparison test was employed. This method controlled for potential Type I errors while identifying variations in group means.

## RESULTS

**Figure 5:**
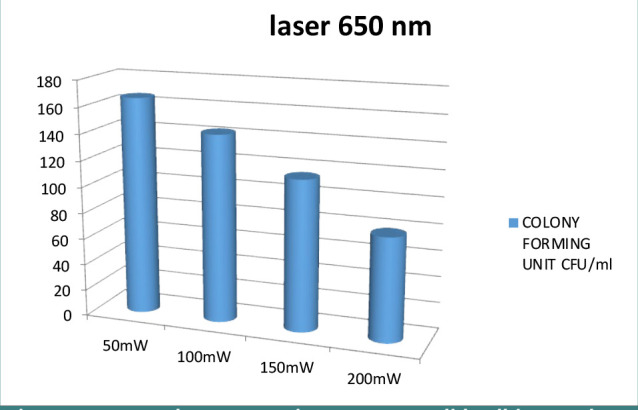
650 nm laser power impact on Candida albicans colony formation

We used various power levels to identify the most effective power setting for inhibiting *Candida albicans*, including 50 mW, 100 mW, 150 mW, and 200 mW. The effectiveness of each setting was assessed based on the CFU/mL (Figure 5). Our findings revealed that the 200-mW setting had the best results inhibiting *Candida albicans* growth.

The optimal inhibition time was 300 seconds, effectively reducing the colony-forming units per milliliter (Table 1). The LSD test was employed to determine the statistical significance of differences among the tested means. In this context, the letters A, B, C, D, and E denote varying levels of significance. The sequence started with the letter A, which indicated the highest significance level, followed by subsequent letters indicating decreasing significance levels. Specifically, the letters B, C, D, and E represented progressively lesser degrees of statistical significance. This approach facilitated identifying and ranking significant and highly significant differences among the tested means at the level of *P* = 0.001.

**Table 1:** Effect of 650 nm diode laser exposure time on the reduction of Candida albicans colony counts at 10-4 CFU/mL concentration

650 nm laser 200 mw	Control	100 Sec.	150 Sec.	200 Sec.	250 Sec.	300 Sec	*P* value
*n*	5	5	5	5	5	5	0.001
Mean × 10^-4^ CFU/mL	299.90	E 283.20	D 223.20	C 172.20	B 132.80	A 83.00	
Median	299.00	283.00	222.00	172.00	133.00	84.00	
Std. Error of Mean	2.05	1.07	1.24	0.86	0.86	0.89	
Std. Deviation	5.3	2.39	2.77	1.92	1.92	2.00	
Minimum	258.10	280.00	220.00	170.00	130.00	80.00	
Maximum	296.20	286.00	227.00	175.00	135.00	85.00	

**Figure 6:**
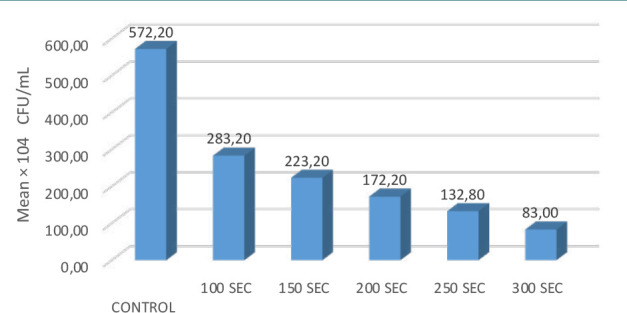
The effect of diode laser (200 mW) on the susceptibility of C. albicans at different times

Figure 6 presents the fungal count values of *Candida albicans* over different time intervals. The data indicates that the percentage of inhibition of *Candida albicans* increased notably at 300 seconds, reaching its peak at this time. Subsequently, there was a decrease in the percentage of inhibition, but this decline was less pronounced at 100 seconds, suggesting that the efficacy of inhibiting *Candida albicans* growth was more pronounced at the 300-second mark compared to the 100-second interval.

Table 2 shows the descriptive and statistical analysis for CFU/mL between the control and the experimental groups using the LSD test. Group 5 (laser and nystatin) had the highest mean significance, followed by Group 4 (laser + MB), Group 2 (antifungal alone), and Group 3 (laser alone) (*P* = 0.001).

**Table 2:** Comparative analysis of *C. albicans* CFU/mL across control and test groups

650 nm at 200 mw, 5 minutes	Control	AF	Laser	L+MB	L+AF	*P* value
*n*	5	5	5	5	5	0.001
Mean × 10^4^ CFU/Ml	299.90	C 69.20	D 76.00	B 45.00	A 22.60	
Median	299.00	70.00	76.5.00	44.00	23.00	
Std. Error of Mean	2.05	1.02	1.58	1.30	0.83	
Std. Deviation	5.3	2.28	1.94	2.72	2.01	
Minimum	258.10	66.00	65.00	43.00	18.00	

AF, antifungal; L, Laser; L+MB, Laser + Methylene blue; L+AF, Laser +antifungal

Table 3 shows multiple comparisons between the control and other groups using Dunnett’s test and found significant differences across groups.

**Table 3:** Comparison between different groups

Multiple comparisons/650 nm at 200 mw, 5 minutes						
(I) Groups	(J) Groups	Mean Difference (I-J)	Std. Error	Sig.	95% Confidence Interval	
					Lower Bound	Upper Bound
AF	Control	489.00	1.61741	.000	480.2878	471.7122
Laser	Control	467.20	1.61741	.000	471.4878	462.9122
L+MB	Control	499.20	1.61741	.000	503.4878	494.9122
L+AF	Control	526.60	1.61741	.000	530.8878	522.3122

AF, antifungal; L, Laser; L+MB, Laser + Methylene blue; L+AF, Laser +antifungal

* The mean difference was significant at the 0.05 level.

**Figure 7:**
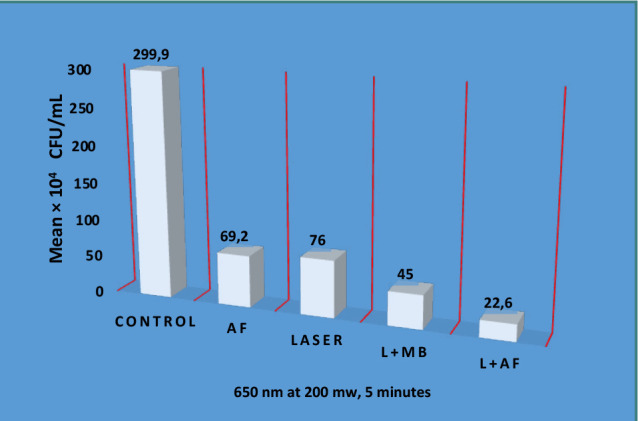
CFU/mL mean percentage across groups AF, antifungal; L, Laser; L+MB, Laser + Methylene blue; L+AF, Laser +antifungal

There was a significant difference in the CFU/mL between the control and the other groups (Figure 7). The highest percentage was found in the control group, followed by the laser-alone, antifungal, and laser and MB groups. The most effective reduction in *C. albicans* was observed in the laser and antifungal combination group.

## Discussion

This in vitro study evaluated the fungicidal effect of a 650 nm diode laser against Candida albicans, the primary pathogen involved in oral candidiasis [19,20]. This condition is increasingly observed in modern clinical settings, often in individuals with compromised immune systems. The presence of these strains contributes to heightened pathogenicity and frequently leads to innate resistance against various antifungal medications, as documented in prior studies [10-12,15,25-32]. Given the rising incidence of resistance to conventional antimicrobials, exploring alternative therapeutic approaches, including the application of laser light, has become imperative [33,34-38]. While the medicinal properties of light have been subjects of investigation for many years, its relatively recent application for antibacterial purposes is noteworthy. This underscores the evolving and innovative nature of research in this field as scientists seek novel strategies to combat antimicrobial resistance and enhance treatment outcomes [39-42].

Studies have investigated the efficacy of diode lasers across the infrared (IR) spectrum at varying wavelengths, especially for the treatment of periodontal disease [43,44]. In clinical practice, we have encountered cases that prove refractory to conventional treatments, including light-based therapies, and even some highly complex cases that do not respond to antibacterial interventions. This observation raises the possibility that the development of these clinical cases may have been further complicated by undetected fungal contamination, particularly an over-colonization of Candida species.

The primary objective of this research was to pioneer laser-based alternatives to antibiotics and other commonly used drugs for controlling fungal infections in the oral cavity. Our investigation explored microbial inactivation through photodynamic therapy or light systems as potential novel therapeutic options for treating oral fungal infections. This became imperative due to the growing prevalence of Candida strains that exhibit resistance to standard antifungal medications and the adverse effects often associated with these drugs [20,21]. These alternatives to conventional medication are gaining importance and necessity in clinical practice. They offer effective treatment with fewer adverse effects and without the limitations imposed by antimicrobial resistance, a challenge frequently encountered with current antibiotics. Indeed, using photodynamic therapy with a photosensitizer to target and eliminate microbes presents an intriguing and promising avenue for further exploration [18,35,45].

Photosensitizers such as methylene blue, tetramethylbenzidine (TB), indocyanine green (ICG), malachite green (MG), or Photogem have been carefully selected for their ability to interact with fungal cells. Moreover, the choice of appropriate light sources is critical to enhancing the efficacy of photodynamic treatment when addressing yeast-related infections [22,26].

The results in Table 1 indicate significant differences in colony-forming units as the exposure time to the treatment increased. These findings align with prior research, suggesting that *C. albicans* could be effectively eliminated when subjected to low-powered light in the presence of an appropriate photosensitizer [46]. This consistency in outcomes underscores the potential efficacy of photodynamic therapy in reducing *C. albicans* populations, mainly when extended exposure times are employed in combination with suitable photosensitizers. Such findings contribute to the growing body of evidence supporting the utility of this therapeutic approach for managing *C. albicans* infections. The mean percentage of colony-forming units in Group 4 (treated with laser and MB) was significantly higher compared to the mean difference between the control group (Group 1) and Group 3 (treated with laser alone), as presented in Table 2.

This phenomenon can be attributed to the unique properties of methylene blue as a photosensitizer. Methylene blue has two absorption peaks at 635 nm and 670 nm wavelengths, encompassing its spectrum from 609 to 690 nm [47]. Various authors, including those referenced in previous studies [33,37,48-50], have utilized methylene blue as a photosensitizer in similar research efforts. Their work has consistently demonstrated the effectiveness of methylene blue-mediated PDT to diminish the viability of *Candida albicans* and other fungal microorganisms. In line with these findings, the results of our study illustrate that PDT can completely stop the growth of *C. albicans*, resulting in a reduction of colony-forming units per milliliter by up to 100%. This outcome is in accordance with other studies where PDT inhibition rates ranged from 74% to 95%. It is noteworthy that our study particularly focused on the use of methylene blue as a photosensitizer [33,37].

These consistent findings across different studies highlight the effectiveness of this approach in targeting and inhibiting the growth of *C. albicans*, further supporting the potential utility of photodynamic therapy as a valuable tool in managing fungal infections. Additionally, in another study, the application of malachite green activated by a 650 nm laser light at a specific dosage also led to a substantial 98.4 percent reduction in CFU/mL [51]. These findings further demonstrate the potential of different photosensitizers and laser wavelengths in effectively targeting and inhibiting the growth of *Candida albicans* [33]. The current study adhered to a standardized protocol that incorporated specific elements, including a 96-well plate, a 650 nm visible light laser, a methylene blue photosensitizer concentration of 100 µg/ml, a 5-minute incubation period, and an irradiation time of 300 seconds. These parameters and variables closely align with other studies of photodynamic therapy aimed at inhibiting microbial activity [33,37], supporting the hypothesis that PDT can serve as an effective antifungal strategy against *Candida albicans*

## Conclusion

Photodynamic therapy was an effective alternative or adjunct to conventional antimicrobial therapies, demonstrating significant antifungal activity against *Candida albicans*. This approach is particularly valuable in clinical settings where superinfections compromise patient outcomes. Unlike antiseptic products such as nystatin, photodynamic therapy offers the advantage of minimizing side effects. The optimal therapeutic effect, combining laser treatment with antifungal agents, was achieved at 300 seconds.

## Personal thanks

The authors acknowledge the support and collaboration of the Institute of Laser for Postgraduate Studies at the University of Baghdad, Baghdad, Iraq, as well as the Basic Science and Microbiology Department at the University of Baghdad, for their invaluable contributions to the clinical and laboratory work.
